# The Epigenome in Multiple Myeloma: Impact on Tumor Cell Plasticity and Drug Response

**DOI:** 10.3389/fonc.2018.00566

**Published:** 2018-12-11

**Authors:** Eva De Smedt, Hui Lui, Ken Maes, Kim De Veirman, Eline Menu, Karin Vanderkerken, Elke De Bruyne

**Affiliations:** ^1^Department of Hematology and Immunology-Myeloma Center Brussels, Vrije Universiteit Brussel, Brussels, Belgium; ^2^Department of Hematology, Tianjin Medical University General Hospital, Tianjin, China

**Keywords:** multiple myeloma, epigenetics, histone methyltransferases, histone demethylases, MM cell plasticity, drug response

## Abstract

Multiple myeloma (MM) is a clonal plasma cell malignancy that develops primarily in the bone marrow (BM), where reciprocal interactions with the BM niche foster MM cell survival, growth, and drug resistance. MM cells furthermore reshape the BM to their own needs by affecting the different BM stromal cell types resulting in angiogenesis, bone destruction, and immune suppression. Despite recent advances in treatment modalities, MM remains most often incurable due to the development of drug resistance to all standard of care agents. This underscores the unmet need for these heavily treated relapsed/refractory patients. Disruptions in epigenetic regulation are a well-known hallmark of cancer cells, contributing to both cancer onset and progression. In MM, sequencing and gene expression profiling studies have also identified numerous epigenetic defects, including locus-specific DNA hypermethylation of cancer-related and B cell specific genes, genome-wide DNA hypomethylation and genetic defects, copy number variations and/or abnormal expression patterns of various chromatin modifying enzymes. Importantly, these so-called epimutations contribute to genomic instability, disease progression, and a worse outcome. Moreover, the frequency of mutations observed in genes encoding for histone methyltransferases and DNA methylation modifiers increases following treatment, indicating a role in the emergence of drug resistance. In support of this, accumulating evidence also suggest a role for the epigenetic machinery in MM cell plasticity, driving the differentiation of the malignant cells to a less mature and drug resistant state. This review discusses the current state of knowledge on the role of epigenetics in MM, with a focus on deregulated histone methylation modifiers and the impact on MM cell plasticity and drug resistance. We also provide insight into the potential of epigenetic modulating agents to enhance clinical drug responses and avoid disease relapse.

## Introduction

Multiple myeloma (MM) is a plasma cell (PC) malignancy that mainly resides in the bone marrow (BM). The malignant PCs produce an excess amount of monoclonal antibodies (M-proteins), detectable in serum and/or urine of the patient. The expansion of malignant cells interferes with the normal function of the BM, resulting in anemia, cytopenia, fatigue, and bone pain (1). The BM microenvironment plays a central role in the pathogenesis of MM. This includes a cellular compartment composed of many different cell types (e.g., fibroblasts, mesenchymal stem cells, osteoblasts, osteoclasts, adipocytes, endothelial cells, myeloid-derived suppressor cells, and macrophages) and a non-cellular compartment, including soluble growth factors (e.g., IL-6, IGF-1, VEGF, bFGF, BAFF, APRIL, and SDF-1), adhesion molecules and exosomes. Functional, bi-directional interactions between the MM cells and the different BM compartments foster not only MM cell survival, proliferation and migration, but also contribute to the development of drug resistance, osteolysis, increased angiogenesis and immune suppression (2–5).

MM accounts for more than 10% of all hematological malignancies and is consistently preceded by a premalignant condition known as monoclonal gammopathy of undetermined significance (MGUS). MGUS evolves to symptomatic myeloma with a rate of 1% per year. Symptomatic myeloma is diagnosed by the presence of ≥10% clonal PCs in the BM, the presence of M-protein in the serum and/or urine and evidence of end-organ damage (including hypercalcemia, renal failure, anemia and bone lesions, commonly referred to as the CRAB criteria) (6). Over the past 15 years, advances in therapy have doubled life expectancy of MM patients using novel agents, including proteasome inhibitors (PIs) (bortezomib and second generation inhibitors like carfilzomib) and/or immunomodulatory drugs (IMiDs) (lenalidomide and pomalidomide), in combination with autologous stem cell transplantation, alkylating agents (melphalan) and/or glucocorticoids (prednisone and dexamethasone) (7). In addition, novel classes of agents were introduced in the treatment regimens including epigenetic modifying agents, such as the histone deacetylase (HDAC) inhibitor panobinostat, and monoclonal antibodies, namely elotuzumab and daratumumab. Unfortunately, despite the important gain in survival, most patients will ultimately relapse and develop non-responsive disease. Myeloma thus remains incurable for the majority of patients, clearly demonstrating the need for novel treatment options.

MM is a genetically and clinically highly complex and heterogeneous disease, reflected by the presence of a high number of non-recurrent genetic defects, a branching pattern of clonal evolution and different patient outcomes. The accumulation of genetic aberrations throughout the disease evolution has an impact on numerous important pathways, thus affecting prognosis and response to treatment. MM patients can be divided into 2 groups based on their karyotype; the hyperdiploid and non-hyperdiploid group. Fifty to sixty percent of MM patients display a hyperdiploid karyotype, characterized by trisomies involving chromosomes 3, 5, 7, 9, 11, 15, 19, and 21 (3). Common non-hyperdiploid defects include monosomy 13, gains of 1p or recurrent translocations involving the immunoglobulin heavy chain (IgH) locus. The most frequent translocations are t(11;14)(q13;q32) and t(4;14)(p16;q32); the former dysregulates the CCND1 gene and the latter upregulates FGFR3 (fibroblast growth factor receptor 3) and MMSET genes. Next to these primary events, MM is characterized by secondary events which occur during disease progression and lead to the formation of different subclones, thus adding to the complexity of the disease. These aberrations include, amongst others, MYC overexpression, mutations in members of the NFkB pathway, activation of oncogenes including RAS family members (NRAS, KRAS, and BRAF) and CCND1; and inactivation of tumor suppressor genes like p53, RB1, CDKN2A, and CDKN2C (3, 8).

Next to the above-mentioned role of the BM microenvironment and genetic alterations in MM pathogenesis, it has become increasingly clear that the epigenetic machinery also plays a crucial role in MM. Like in other cancers, the epigenetic landscape is completely disturbed in MM cells. Intergenic regions are often hypomethylated leading to genomic instability, while promoter-associated CpG islands of tumor suppressor genes and miRNAs are hypermethylated and/or deacetylated leading to a loss-of-function (9). Moreover, genetic defects in-, and overexpression of several chromatin modifying enzymes have been described in MM. Importantly, these epimutations are often associated with genomic instability, emergence of drug resistance, MM progression and short progression free survival. In addition, the epigenetic machinery is also linked with MM cell plasticity, driving the differentiation of the malignant cells to a less mature and drug resistant state (10). Recently, it has become clear that next to the well described importance of aberrant DNA methylation and histone acetylation in MM, abnormal histone methylation also plays an important role in MM pathogenesis, as evidenced by the high number of mutations found in histone methyltransferases (HMTs) and -demethylases (HDMs) (11, 12). Here we review the current state of knowledge on the role of epigenetics in MM, with a focus on the deregulated HMTs and HDMs and their contribution to clonal heterogeneity, plasticity and drug response in MM.

## DNA Methylation

DNA methylation is by far the most studied epigenetic modification and has a profound impact on genome stability and gene expression patterns. A methyl group is added to the carbon-5 position of a cytosine in a cytosine-phosphate-guanine (CpG) dinucleotide, resulting in 5-methylcytosine (5 mC) (13). DNA methylation at CpG dinucleotides has historically been associated with stable and permanent gene repression. However, it is now well-known that DNA methylation is a reversible process. New DNA methylation patterns are established by the *de novo* DNA methyltransferases DNMT3A and DNMT3B, while DNMT1 is responsible for maintaining methylation patterns upon replication (13). In contrast, demethylation is initiated by the TET (Ten-eleven translocation) enzymes; TET1, TET2, and TET3. These enzymes use molecular oxygen as a substrate to convert 5mC to 5-hydroxymethylcytosine (5hmC) and 5hmC to 5-formylcytosine (5fC) and 5-carboxycytosine (5caC). Thymine-DNA glycosylase (TDG)-mediated base excision repair (BER) of 5fC and 5caC can then regenerate unmethylated cytosine nucleotides (active demethylation). Moreover, the oxidized states of cytosine hinder DNMT1 binding, leading to a loss of methylation during replication (passive DNA methylation) (14).

In healthy cells, around 60–80% of the CpGs in the human genome are methylated. These methylated CpGs are mainly located in gene bodies and genome-stabilizing repetitive elements. In contrast, around 10% of the CpGs are grouped in CG dense regions called CpG islands. These islands are mostly located in close proximity of transcription start sites and are often unmethylated, thus permitting gene expression. In cancers cells, including MM cells, global DNA hypomethylation and gene-specific promoter hypermethylation is often observed (15). In MM patients, the repetitive elements LINE-1, Alu, and SAT-a are hypomethylated compared to healthy controls, correlating with genomic instability, disease progression and poor prognosis (16–18). Next to this global hypomethylation, MM is also characterized by the silencing of several cancer-related genes through hypermethylation, including but not limited to p73, p53, p15, p16, E-CAD, DAPK1, BNIP3, RB1, DIS3, CDKN2A, and CDKN2C (19). Notably, promotor hypermethylation of p16, BNIP3, DAPK1, and E-CAD has furthermore been associated with poor prognosis (19–23). Only very recently, we demonstrated that RASSF4 is also silenced through promotor methylation during MM progression, correlating with a bad prognosis. RASSF4 is a member of the Ras-Association Domain Family (RASSF), responsible for mediating the anti-tumoral effects of RAS. We found RASSF4 loss to unleash the pro-mitogenic activity of RAS in MM. Treatment with epigenetic modifying agents restored RASSF4 expression, thereby sensitizing MM cell to the clinically relevant MEK1/2 inhibitor trametinib (24). Although rare, promotor hypomethylation also plays a role in (early) disease pathogenesis. The NOTCH ligand JAG2 for example was shown to be overexpressed in malignant PCs from MGUS and MM patients. This JAG2 overexpression was due to hypomethylation of the JAG2 promoter and enhanced the secretion of the growth factors IL-6, VEGF, and IGF-1 in stromal cells (25). In addition, the expression level of the so-called breast cancer resistance protein (BCRP/ABCG2), a membrane drug efflux pump, was demonstrated to be increased upon chemotherapy through promotor demethylation, thus promoting drug resistance (26).

Importantly, genome-wide analysis of DNA methylation patterns revealed that these patterns change during MM progression. In 2011, Walker et al. published genome-wide methylation microarray data from different MM stages, showing that hypomethylation is already present in the early stages of MM development, and the methylation levels further decrease during disease progression. In contrast, gene-specific hypermethylation is rather a rare event (17, 27). Nevertheless, this promotor methylation increases during MM progression, reaching its maximum in the plasma cell leukemia stage (PCL) (17). Walker et al. furthermore reported that the highest frequency of hypermethylated genes was present in the t(4;14) translocation subgroup, present in 15-20% of the MM population and associated with a bad prognosis (17, 28). Moreover, an overlap of hypermethylated genes was found between the t(4;14) subgroup and PCL samples, further suggesting the contribution of the gene-specific hypermethylation to disease progression and aggressiveness (17). Importantly, in B cell tumors, DNA hypermethylation is mainly present in polycomb repressed/bivalent regions. In normal precursor cells, these regions are often hypomethylated. In B cell malignancies, however, the H3K27me3 marks are often replaced by DNA methylation, referred to as “Polycomb repression-associated DNA methylator phenotype” or PRAMP. This epigenetic switching is suggested to reduce regulatory plasticity of key regulatory genes (29–32). In the study of Aggire et al., the DNA methylome was recently analyzed on a broader level, including promotors, gene bodies, and intergenic regions of normal PC, MGUS, and MM patients. Interestingly, they found that hypermethylation in MM patients is not only restricted to promotor associated CpG islands, but is also present in intronic enhancer regions of B cell specific genes and transcription factors leading to the downregulation of B cell associated transcription factors such as PAX5, BATF, and STAT5 (33). The exact mechanisms underlying these aberrant methylation patterns in MM remain to be elucidated. A possible explanation might be the downregulation of miR-29b, which targets DNMT3A and DNMT3B, resulting in an aberrant methylation profile (34, 35). In addition, increased levels of DNMT1 and DNMT3A have been reported and miR-22 upregulation results in the inhibition of TET2. Lastly, mutations in methylation modifying enzymes including TET1/2/3, IDH1/2 and DNMT1/3A/3B have been described in MM (10).

DNA methyltransferase inhibitors (DNMTi) are often used to revert aberrant DNA methylation patterns in cancer cells. Two DNMTi commonly used in preclinical and clinical settings are the cytidine analogs 5-azacytidine (AZA) and 5-aza-2′deoxycytidine (decitabine; DAC). Upon incorporation into DNA, these analogs will covalently bind DNMTs resulting in the degradation of the enzymes and thus passive demethylation. In addition, these agents mediate direct cytotoxic effects, as evidenced by the induction of a DNA damage response (36). AZA and DAC are currently approved for the treatment of myelodysplastic syndromes (37). In MM, we and others have also confirmed the anti-myeloma activity of AZA and DAC and this both *in vitro* and *in vivo* (38–42). In short, we showed that DAC induces DNA damage in MM cells, resulting in cell cycle arrest and apoptosis. Moreover, using the 5T33MM model, we demonstrated that DAC also displays potent *in vivo* anti-myeloma activity. Mechanistically, *in vivo* DAC treatment was found to deregulate genes involved in immune regulation, regulation of gene expression and metabolism (40). DNMTi are also valuable prognostic tools. We and others constructed gene expression (GEP)-based risk scores to predict sensitivity of MM cells to pan-DNMTi. In 2012, Moreaux et al. developed a gene-expression based DNA methylation score to predict DAC sensitivity in MM cells. The score allowed the identification of high-risk MM patients who could benefit from DNMTi treatment (43). We recently validated this work *in vivo* using the murine 5T33MM model. By analyzing the *in vivo* transcriptional response of 5T33MM cells toward DAC and the histone deacetylase inhibitor (HDACi) quisinostat, we identified a DNA methylation and histone acetylation score predictive for overall survival of MM patients. A high score correlated with a highly proliferative and immature phenotype of MM cells and a bad prognosis (39). Currently, clinical trials are ongoing with DNMTi as monotherapy or combined with lenalidomide or dexamethasone in MM (44).

## Histone Modifications

Chromatin consists of repeating units of nucleosomes, each consisting of 146 bp of DNA wrapped around a histone octamer composed of 4 histones (H2A, H2B, H3, H4) and the linker histone H1. Histone proteins have N-terminal tails protruding from the nucleosome, which are prone to reversible modifications, including methylation, acetylation, phosphorylation, ubiquitination, sumoylation and deamination at lysine, arginine, threonine, and serine residues. These so-called post-translational histone modifications alter the structure and density of the chromatin, which in turn influences the accessibility of the DNA for the transcription machinery and many other DNA related processes like DNA repair, replication, and recombination (45, 46).

### Histone Acetylation

One of the best studied post-translational histone modifications is histone acetylation. This process is mediated by two different enzyme families: histone acetyltransferases (HATs) and histone deacetylases (HDACs). HATs transfer an acetyl group from the donor acetyl-CoA to lysine residues of histone tails. This leads to a neutralization of the positive charge of the histones and a more open configuration and therefore correlates with active transcription. In contrast, HDACs will remove these charge-neutralizing acetyl groups, leading to a more condensed chromatin structure and transcriptional silencing. Thus far, the HDAC family consists of 18 members, divided into four classes. The Zn^2+^-dependent HDACs include Class I (HDAC 1–3 and 8), IIa (HDAC 4,5,7, and 9), IIb (HDAC 6 and 10) and IV (HDAC 11), and the NAD-dependent HDACs form the class III enzymes (SIRT1 to 7). Apart from histones, these enzymes also have several other, non-histone substrates including transcription factors such as p53, DNA repair enzymes and chaperones (47). In MM, the expression of several HDAC members (mainly class I HDACs) is upregulated, correlating with a poor prognosis (48). As a result, diverse pan-HDAC inhibitors have been (pre)clinically tested for their therapeutic value in MM. In the PANORAMA trials, the effect of the pan-HDACi panobinostat in combination with bortezomib and dexamethasone was investigated in relapsed and/or refractory MM. Results showed a significant improvement in the progression free survival of patients who received prior treatment with both bortezomib and an IMiD. (49, 50). Based on these results, panobinostat was FDA approved for the treatment of relapsed/refractory MM patients, who received 2 prior treatment regimens including bortezomib and IMiDs (51). However, panobinostat was only approved with a Risk Evaluation and Mitigation Strategy due to the high risk of high grade (non)hematological toxicities, thus limiting the broad application of this pan-HDACi. The use of more selective HDAC inhibitors could reduce the rather severe side effects observed upon panobinostat treatment. A possible candidate is HDAC6, which is involved in autophagy-mediated degradation of misfolded proteins. Aggrosomal degradation is an alternative to the proteasome for protein degradation. Blocking both pathways using bortezomib or carfilzomib and the HDAC6 selective inhibitor ricolinostat (ACY1215) synergistically induced anti-MM effects both *in vitro* and *in vivo* (52–54). A phase I/II clinical trial recently showed ricolinostat to be well tolerated in combination with bortezomib and dexamethasone in relapsed and refractory MM patients, with an overall response rate of 29 and 14% in respectively all patients and bortezomib-refractory patients (55). Moreover, ongoing studies are currently also evaluating ricolinostat in combination with IMiDs and dexamethasone in relapsed and refractory patients (44). In the phase Ib dose-escalation study examining ACY-1215 in combination with LEN-DEX (ACE-MM-101), the combination was also found to be well-tolerated and preliminary assessment showed an overall response rate of 55% (56). The results from these early phase studies indicate clinical benefit of selective HDAC6 inhibition, but confirmation is warranted in phase III studies. Next to the deregulated expression of HDACs, mutations in the HATs EP300 and CREBBP were also identified in MM patients. Interestingly, the frequency of CREBBP mutations further increased in relapsed patients, thus suggesting a role in drug resistance (11).

### Histone Methylation

**Figure 1:**
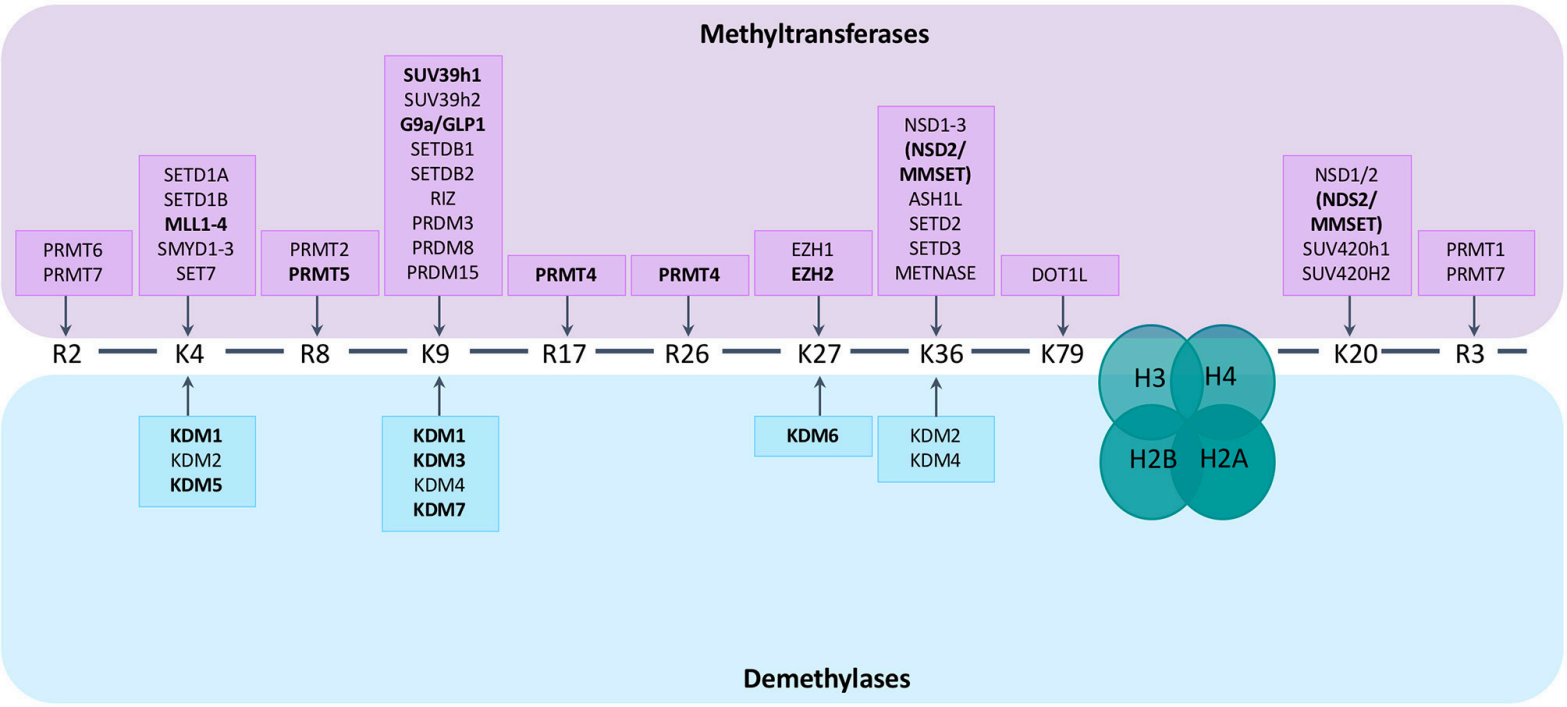
Histone methyltransferases and demethylases and their targets. Lysine (K) and arginine (R) residues of histone 3 (H3) and histone 4 (H4) are shown. Histone methyltransferases and demethylases are grouped based on the specific histone tail residue that they target. Lysine residues can be mono-, di- or tri-methylated, arginine residues can be mono- and di-methylated. Enzymes known to play a role in MM pathogenesis are depicted in bold.

Histone methylation is a more complex post-translational histone modification than histone acetylation. Methylation mainly occurs at lysine or arginine residues of histones H3 and H4 and is mediated by HMTs and HDMs. The effect of the addition of a methyl group on gene expression depends on the residue that is methylated and the number of added groups (mono-, di- or tri-methylation for lysine residues, mono- and di-methylation for arginine residues) (57, 58). In general, methylation of H3K4, H3K36, and H3K79 is associated with gene activation, while H3K9, H3K27, and H4K20 methylation is associated with gene silencing. Adding to the complexity, the outcome of methylation marks is furthermore dependent on the genomic distribution (57). There are over 30 lysine histone methyltransferases (KMT) belonging to 8 families (KMT1-8). Based on their structure they can be divided into 2 main classes; one class containing a SET domain, the other class containing a DOT1L domain (58). Protein arginine N-methyltransferases (PRMTs), responsible for the addition of methyl groups to arginine residues of histone tails, can be subdivided into 3 subtypes (type I–III) based on the type of arginine methylation that is formed: ω-NG, NG–asymmetric dimethylarginine (ADAM), ω-NG,N'G–symmetric dimethylarginine (SDMA), and ω-NG- monomethylarginine (MMA). There are currently 9 PRTMs described. The enzymes responsible for demethylation of the lysine residue are the histone lysine demethylases (KDMs). These KDMs can be divided into 2 subgroups based on their mechanism of action. KDM1 family members depend on flavine adenine dinucleotides (FAD) as co-factors for the demethylation of H3K4me1/2 or H3K9me1/2 residues. The other KDM members contain a Jumonji C (JmjC) domain and depend on Fe(II)- and 2-oxoglutarate oxygenases as co-factors. They are further subdivided into different classes (KDM2, KDM3, KDM4, KDM5, KDM6 and other, less studied enzymes like KDM7) (59). Less is known regarding methylarginine demethylases (RDMs). It was recently shown that some members of the KDM family, like KDM3A and KDM6B, can demethylate arginine residues aswell (60). Next to histones, the histone-methylation enzymes also target non-histone proteins, thereby influencing important cellular signal pathways, including NFkB, RAS, PI3K/Akt, Wnt/β-catenin, P53 and ERα pathways (61). An overview of the enzymes catalyzing histone (de)methylation and their histone targets is provided in Figure 1.

Aberrant expression or mutations of HMTs (writers), HDMs (erasers) and methyl-binding proteins (readers) are increasingly linked with cancer (10, 12, 62). In MM, whole-exome sequencing analysis of 463 newly diagnosed MM samples revealed that mutations in a wide range of epigenetic regulators, so-called epimutations, are present in 50% of the MM patients. When comparing sequencing data from diagnosis with later stages of the disease, an increase in the presence of these epimutations was observed, suggesting a role in MM progression. In addition, a higher mutational burden in histone methyltransferases and DNA methylation encoding genes (such as NSD1, MLL2-3, SETD2, and DNMT3A) was observed upon relapse, indicating a role in drug resistance to current therapies (11, 12, 63). Below we will discuss the recent findings on the role of HMTs and HDMs in MM pathogenesis.

## Histone Methylation Enzymes and Multiple Myeloma

### Histone Methyltransferases in Multiple Myeloma

One of the best studied HMTs in MM is the lysine methyltransferase **MMSET** (also known as WHSC1 or NSD2). The t(4;14) translocation, resulting in the fusion of MMSET to the IgH locus, results in the overexpression of MMSET and FGFR3 and is present in 15–20% of patients with MM. This translocation is associated with a shorter event-free and overall survival, but bortezomib treatment is able to overcome this poor prognosis (64). Moreover, an MMSET gain-of-function mutation E1099K has been identified in lymphoid malignancies, including MM (65). MMSET mainly catalyzes the dimethylation of H3K36 and di- and trimethylation of H4K20. In MM, MMSET overexpression results in the accumulation of H3K36me2 levels, causing transcriptional activation of oncogenes and promoting oncogenic transformation of primary cells (45, 58, 66, 67). In addition, MMSET overexpression results in the genome-wide redistribution of the SET lysine methyltransferase EZH2 and the associated H3K27me3 mark (68). Gene expression profiling and pathway analysis revealed affected genes to be mainly involved in cell cycle (e.g., Cyclin E2), apoptosis and p53 pathway (e.g., BAX and Bcl2), DNA repair (e.g., ATM and GADD45A), and integrin mediated signaling (e.g., CDC42) (66). In concordance, MMSET loss of function experiments indicated anti-MM effects such as cell cycle arrest and induction of apoptosis (66, 69). More recently, MMSET was shown to methylate Aurora kinase A (AURKA) resulting in the proteasomal degradation of p53, thus increasing proliferation in solid tumors (70). Moreover, MMSET was also shown to act as a co-activator for the NFkB pathway (71). Furthermore, MMSET was demonstrated to interact with epigenetic repressors such as sin3a, HDAC1-2 and the lysine specific H3K4 demethylase LSD1/KDM1A, thus forming transcriptional repressor complexes. For example, repression of the microRNA miR-126 by a complex formed by MMSET, KAP1, and HDACs was shown to increase c-MYC levels, thus stimulating MM proliferation (72, 73). Finally, MMSET has been shown to play a role in the DNA damage response. In mammals, MMSET is recruited to double strand breaks, where it mediates methylation of H4K20, thus stimulating p53-binding protein (53BP1) recruitment and DNA repair (74, 75). In myeloma, MMSET high cells were shown to repair melphalan induced DNA damage at an enhanced rate and continued to proliferate, while MMSET low cells accumulated DNA damage and entered cell cycle arrest. Moreover, MMSET silencing increased sensitivity toward melphalan treatment *in vivo* (76). Together, these findings suggest that the enhanced DNA damage repair potential of MMSET overexpressing MM cells is a possible resistance mechanism, making these MM patients less sensitive toward treatment with DNA-damaging agents. This notion is further supported by the observation that t(4;14) patients experience rapid relapse upon treatment with DNA damage-inducing agents such as melphalan (64, 77). Collectively, MMSET represents an interesting therapeutic target in MM. So far, however, no potent specific MMSET inhibitors are commercially available yet. LEM-06 is a small molecule inhibitor of MMSET, but lacks potency and efficacy (IC50 = 800 uM). Recently, by means of high-throughput screening, 5 possible MMSET inhibitors were identified. Further work is needed in order to validate these inhibitors in MM (78).

The **enhancer of Zeste Homologue 2 (EZH2)** is the catalytic subunit of the polycomb repressor protein complex 2 (PRC2). EZH2 can mediate the 3 methylation states of H3K27, however, the most investigated action of EZH2 is gene silencing through trimethylation of H3K27 (H3K27me3) (79, 80). EZH2 mediated trimethylation of H3K27 recruits canonical PRC1 and other corepressive factors to the DNA by serving as a docking site. PRC1 will then catalyze monoubiquitylation of H2A on lysine 119 (H2AK119), leading to a more compact state of the chromatin associated with gene silencing (80, 81). In addition, EZH2 can also methylate non-histone proteins like STAT3, leading to enhanced STAT3 activity and an increase in tumorigenic potential of glioblastoma stem-like cells (82). Lastly, EZH2 also functions as a co-activator for several transcription factors, thus activating pathways such as the NFkB signaling cascade (83).

Importantly, several studies identified polycomb group genes, including EZH2, as crucial factors mediating stem cell pluripotency and self-renewal (79, 84, 85). EZH2 overexpression is described in various solid cancers and hematological malignancies, including lung, breast, and pancreatic cancer and diffuse large B cell lymphoma, and is often linked to a more aggressive phenotype and unfavorable prognosis (83, 86). Moreover, activating EZH2 mutations disturb normal B cell differentiation and have been linked to the development of diffuse large B cell and follicular lymphomas (86). However, in some cancers like myelodysplastic syndrome, mutational inactivation of EZH2 is linked to a bad prognosis, indicating that the function of EZH2 in cancer may be cell-context dependent (87).

Recently, EZH2 has also become a hot topic in MM, as evidenced by the high number of publications that emerged during the last years. Gene expression profiling revealed an upregulation of EZH2 levels during MM progression, together with an elevated expression in the high risk proliferative molecular subgroup (86, 88, 89). EZH2 overexpression is associated with an inferior progression-free and overall survival, and this independently from the treatment used (86, 90). Notably, EZH2 overexpression in MM has been linked to stimulation of the IL-6R, c-MYC activation, and miR26a downregulation (10). In line with the EZH2 overexpression, Kalushkova et al. identified a common silenced gene signature in MM patients enriched for H3K27me3-regulated polycomb target genes (89). Later on, Agarwal et al. elaborated on this finding by performing a genome-wide profiling study investigating H3K27/4me3 marks in MM. Again, a common set of active (H3K4me3 enriched) and inactive (H3K27me3 enriched) genes specific for MM samples was identified. Importantly, the MM unique H3K27me3 mediated gene silencing was found to correlate with disease progression and prognosis (91). More recently, Binder et al. identified a IL6/STAT3-induced long non-coding RNA (lncRNA) termed STAiR18 that was shown to be associated with H3K27me3, suggesting that STAiR18 might be an epigenetic regulator involved in transcriptional silencing in MM (92). In support of this, accumulating evidence has revealed that some lncRNAs can operate as an interface between the epigenetic modification machinery and DNA, enabling the recruitment of chromatin regulators to specific genomic loci (93, 94). A prominent example of such an epigenetic-related lncRNA is HOX transcript antisense RNA (HOTAIR). HOTAIR is well-known to influence chromatin compactness by serving as a molecular scaffold for PRC2, thereby recruiting and affecting PRC2 occupancy on genes genome-wide (95, 96). Importantly, HOTAIR overexpression has been reported in several solid tumors and hematological malignancies, including AML and diffuse large B cell lymphoma, and has been positively correlated with initiation, progression, drug resistance, and poor prognosis (95–99). In MM, however, no aberrant expression of HOTAIR in MM cells so far has been reported (100). Moreover, HOTAIR circulating levels in MM patients were found to be even lower than healthy controls (101). Nevertheless, Binder et al. showed similar enrichment of HOTAIR and STAiR18 by H3K27me3 pulldown in IL-6-treated INA-6 cells. Hence, further studies investigating STAiR18 and/or HOTAIR binding with PRC2 in MM cells are of interest (92). Lastly, EZH2 was also reported to be involved in MM associated bone disease. Previously, it was shown that myeloma cells induce the transcriptional repressor GFI1 in osteoblast precursors, resulting in RUNX2 silencing and suppression of osteoblast differentiation. In a later study, this GFI1-mediated RUNX2 silencing was shown to be dependent on the recruitment of HDAC1, LSD1 (KDM1A), and EZH2 (102). Hence, pharmacological EZH2 inhibition using the EZH2 specific inhibitors UNC1999 and GSK343 induced anti-MM effects by reactivating genes involved in differentiation, cell cycle and apoptosis. Pawlyn et al. also demonstrated the anti-MM effect of 2 additional EZH2 inhibitors (EZH2i), namely EPZ005687 and UNC2400, using HMCL and primary samples derived from heavily pretreated patients. Of note, while a previous study demonstrated an increase in EZH2i sensitivity in MMSET-overexpressing MM cells, Pawlyn et al. found no correlation between high MMSET levels and EZH2i sensitivity (68, 103). This discrepancy should be further investigated. More recently, Alzrigat et al. showed that UNC1999 restores the expression of miR-125a and miR-320c, causing the subsequent downregulation of MM-associated oncogenes, such as IRF-4, Xbp-1, Blimp-1, and c-MYC (104). Moreover, EZH2 mRNA expression levels were found to inversely correlate with miR-29b levels and EZH2 targeting restored miR-29b expression levels and downregulated MM promoting factors like CDK6, MCL-1, HDAC4, and DNMT3A/B (105). The above clearly supports the oncogenic role of EZH2 in MM and its potential use as a therapeutic target. Different EZH2i, such as GSK-926, GSK-343, EPZ-005687, EPZ-6438 (tazemetostat), EI1 and CPI-169, are currently under clinical investigation in solid tumors and in lymphomas, both as single agents and in combination strategies. One of the most investigated inhibitors is EPZ-6438, which showed promising results in clinical trials in lymphoma patients with minimal toxicity problems (79, 106). In MM, disappointing results of a clinical trial investigating the use of GSK2816126 as a single agent so far halted the further development of this agent. However, the identification of relevant biomarkers could improve the clinical benefit of these agents. Concerning this, Herviou et al. recently created a gene-expression based EZ-score, enabling the prediction of EZHi-sensitivity in HMCL and primary MM. The development of such tools could facilitate the identification of patients who could greatly benefit from EZH2i treatment (107).

The nuclear KMT1 members **G9a** (EHMT2) and **GLP** (G9a-like protein/EHMT1) mediate mono-, di- and trimethylation of H3K9. G9a and GLP are highly homologous interaction partners and their binding seems to be crucial for their methyltransferase activity, especially *in vivo*. G9a furthermore acts as a scaffolding protein via its ankyrin-repeats containing domain and interacts with other chromatin-associated proteins like heterochromatin protein 1 (HP-1). This will lead to the subsequent recruitment of DNMT1, resulting in methylation of nearby DNA sites, thus reinforcing transcriptional silencing (108). Notably, G9a expression was shown to mediate the repression of oct-3/4 genes linked to pluripotency and thus plays a crucial role in embryogenesis. Next to histone proteins, G9a/GLP also methylate non-histone targets such as p53 and SIRT1 (109). Overexpression of G9a has been reported in different cancers, correlating with tumor suppressor silencing (such as p53, CDH1, RUNX3, and E-cadherin), metastasis and a worse prognosis (108, 110–112). Importantly, G9a was shown to be upregulated in cancer under hypoxic conditions, resulting in the downregulation of HIF-1α responsive genes and increased cell motility and migration, thus demonstrating a key role for G9a in stimulating cell survival under hypoxic stress (95, 100). Two of the best described G9a/GLP inhibitors are BIX-01294 and UNC0638. Interestingly, both pharmacological and genetic G9a/GLP targeting have been shown to inhibit proliferation and migration of cancer cells and is often associated with the induction of autophagy and the re-expression of tumor suppressor genes (108, 113–115). In MM, GLP was found to be upregulated in smoldering myeloma patients compared to normal BM samples (108, 110). Moreover, using a siRNA screening approach, G9a was identified as a potential target in hematological malignancies such as acute lymphoblastic leukemia, acute myeloid leukemia (AML), lymphoma and MM cell lines. In concordance, BIX-01294 and UNC0638 were shown to inhibit proliferation and induce apoptosis in these cell lines (116). These data indicate a potential role for G9a/GLP targeting in MM patients.

The KMT1 **SUV39H1** was also found to be differentially expressed between normal BM PCs and MM PCs. SUV39 mediates the trimethylation of H3K9, which is a binding site for the adaptor molecule HP-1 (117, 118). This repressive mark is associated with the silencing of tumor suppressor genes in AML and SUV39H1 inhibition was shown to restore the expression of the epigenetically silenced p15INK4B and E-cadherin genes in AML cell lines (117, 119). SUV39H1 was also found to bind to a central repression domain in RUNX1 (also known as AML1), a frequently disturbed gene in AML (120). In MM, high SUV39H1 levels are associated with a bad prognosis. Knock down experiments resulted in a decrease in proliferation and an increase in apoptosis, ROS production, and DNA damage. The SUV39H1 inhibitor chaetocin also exhibited anti-MM effects both in HMCL and primary samples (121). These results identify SUV39H1 as a possible target for MM therapy.

The KMT2 or **MLL** (mixed lineage leukemia) family members exhibit enzymatic activity toward H3K4, thus promoting transcriptional activation (122). The MLL members MLL1-5 were shown to be mutated in up to 7% of MM patients. However, these mutations did not have an impact on progression free nor overall survival (12). Hence, the functional role of these methyltransferases in MM still needs to be elucidated.

**PRMT5** is a type II arginine methyltransferase that catalyzes the symmetric methylation of histones and non-histone proteins on arginine residues. PRMT5-mediated histone arginine methylation is involved in transcriptional repression and activation in a context dependent manner (123). Moreover, PRMT5 is overexpressed in hematological and solid cancers (124). PRMT5 is functionally involved in differentiation, proliferation, homologous recombination and cell migration (124–126). Silencing or inhibition of PRMT5 appears to have anti-tumor effects in several cancers, including mantle cell lymphoma, mixed lineage leukemia and colorectal cancer (127–129). Gulla et al. recently described PRMT5 as a prognostic factor and therapeutic target in MM. PRMT5 was found to be upregulated in MM patients compared to healthy controls and this was associated with a worse clinical outcome. Moreover, BM stromal cell-conditioned medium was shown to increase PRMT5 expression in MM cells. PRMT5 targeting using both siRNA and the recently developed PRMT5 inhibitor EPZ015666 negatively affected cell cycle progression and induced apoptosis in HMCL and primary samples, even in the presence of BM stromal cells or BM stromal cell-conditioned medium. Moreover, oral administration of EPZ015666 also decreased tumor burden in a xenograft mouse model. In contrast to the previously described role of PRMT5-mediated p53 methylation in lymphoma pathogenesis, the observed anti-MM effects of PRMT5 inhibition were found to be p53 independent (130). Instead, TRIM21 was identified as a PRMT5 binding partner in MM cells, resulting in TRIM21-dependent inhibition of the canonical NFkB signaling pathway (131).

The type I **PRMT4**, also known as coactivator-associated arginine methyltransferase 1/CARM1, mediates methylation of H3R2me2a, H3R17me2a, H3R26me2a, and non-histone proteins, thus functioning as a transcriptional activator. Deregulated PRMT4 expression can be observed in various malignancies, including breast and prostate cancer (62). In 2017, Drew et al. investigated for the first time the role of PRMT4 in MM pathogenesis. EZM2302, a selective PRMT4 inhibitor, was shown to exhibit anti-MM effects both *in vitro* and in a myeloma xenograft setting (132). Similarly, Nakayama et al. demonstrated *in vitro* anti-proliferative effects for the PRMT4 specific inhibitor TP-064 in MM (133).

### Histone Demethylases in Multiple Myeloma

**KDM1A**, also known as LSD1, functions as a transcriptional repressor by demethylating H3K4 mono- and dimethylation. However, under specific conditions, KDM1A can also remove methyl groups of H3K9 mono- and dimethylation marks which results in transcriptional activation. Moreover KDM1A also associates with other epigenetic regulator complexes such as the NuRD complex, SIRT1, CoREST/HDAC, MMSET and the SIN3A/HDAC complex (72, 134). Adding further to the complexity, KDM1A also targets non-histone proteins (135, 136). An interesting non-histone target of KDM1A is the tumor suppressor p53. KDM1A will inhibit p53 function, thereby promoting apoptosis (136). KDM1A overexpression has been reported in several solid tumors and hematological malignancies and has been linked to a bad prognosis (137–139). Inhibition of KDM1A in cancer cells induces cell cycle arrest and a decrease in migration and invasion potential, as shown in different *in vitro* studies. In ovarian cancer for example, LSD1 knock out reduced proliferation and increased sensitivity toward cisplatin (136, 140–143). Based on these recent findings, 3 different KDM1A small molecule inhibitors, namely tranylcypomine, GSK-LSD1, and ORY-1001, are currently in phase I/II clinical studies for AML and small cell lung carcinoma (140). The role of KDM1A/LSD1 in MM is still controversial. In line with the above described studies, higher expression levels of LSD1 were found in patients with symptomatic MM and PCL compared to less aggressive MM states. LSD1 knockdown reduced migration, invasion and wound healing in MM cell lines, together with a decrease in E- and N-cadherin and vimentin levels. These results indicate that LSD1 inhibition negatively impacts epithelial-mesenchymal transition in MM. LSD1 targeting was also found to inhibit osteoclastogenesis and to increase MM sensitivity toward HDAC inhibitors (144). Furthermore, as mentioned above, LSD1 forms a corepressor complex with MMSET in MM, thus further supporting the oncogenic function of LSD1 in MM (72). However, more recently, Wei et al. showed that germline mutations in KDM1A play a role in predisposition toward MM development. Siblings with familial early onset MM were found to harbor truncating mutations in the KDM1A gene. This higher mutation level was also observed in non-familial MM patients compared to controls. Moreover, KDM1A was found to be downregulated during MM development, with lower KDM1A levels in MGUS and MM compared to normal PC samples. Of interest, KDM1A mutated cells were enriched for MYC target genes. In concordance, the KDM1A inhibitor GSK-LSD1 was demonstrated to promote the development of MGUS in mice as evidenced by the expansion of the PC population, a secondary immune response and an increase in the amount of detectable serum M-protein. KDM1A inhibition furthermore increased proliferation of the MM cell line U266 and primary MM samples (145). Together, these data support a tumor suppressive role for KDM1A in MM. In agreement, Kerenyi et al. found KDMA1 to be necessary for normal hematopoietic differentiation, by repressing enhancers and promotors of stem and progenitor cell genes. The authors observed a decrease in the formation of white and red blood cells upon LSD1 knockout (146). Thus, it is clear that further efforts are needed to elucidate the role of KDM1A/LSD1 in MM.

The **KDM3/JMJD1C** lysine demethylase family consists of 3 members; KDM3A, KDM3B, and JMJD1C. Under normal circumstances, KDM3A and KDM3B mediate the demethylation of H3K9me1/2 and have a role in spermatogenesis (147). In addition, KDM3A is also involved in stem cell renewal and adipogenesis (148). Several studies show the involvement of KDM3A in cancer (149–151). Overexpression of KDM3A in solid tumors, such as colorectal and breast cancers, correlates with a bad prognosis (152, 153). Recently, Ohguchi et al. described the importance of the KDM3A-KLF2-IRF4 axis in MM cell survival and homing. KDM3A levels were demonstrated to be increased in MGUS and MM patient samples compared to normal PCs, indicating a role in tumor initiation. KDM3A knockdown resulted in clear anti-MM effects, both *in vitro* and *in vivo*. Gene expression profiling of cells transduced with shRNA for KDM3a revealed a downregulation of KLF2 and IRF4. KLF2 is a transcription factor belonging to the Kruppel zinc finger family and plays a role in supporting normal B- and PC functions (154–156). IRF4 is a PC specific transcription factor which is activated during PC maturation and has been shown to be essential for MM oncogenesis (157–159). Thus, in MM cells, KDM3A maintains KLF2 and IRF4 expression and hence survival through H3K9 demethylation. Only very recently, Ikeda et al. demonstrated that KDM3A is upregulated in MM cells cultured in chronic hypoxic conditions and knock down of this KDM induced apoptosis under these circumstances. The hypoxia-mediated KDM3A upregulation was found to be controlled by the transcription factor HIF1α and was shown to induce expression of the long noncoding RNA MALAT1, resulting in the upregulation of glycolytic genes and anti-apoptotic pathways. Interestingly, MALAT expression is also upregulated during MM progression (160, 161). These results identify the HIF1α-KDM3A-MALAT1 as a potential target in the hypoxic MM cell niches. In conclusion, both studies demonstrate the oncogenic function of KDM3A in MM and underline the importance of the development of a specific KDM3A inhibitor (162). Notably, KDM3A has a different structure then other KDM members, which could facilitate the development of specific small molecule inhibitors and limit off target effects (155).

**KDM5** (JARID1) family members (KDM5A-D) mediate the removal of methyl groups from H3K4me1-3, with the highest affinity toward H3K4me3. They function as transcriptional repressors and often associate with other repressors such as HDACs and HMTs. Overexpression of KDM5A and KDM5B is described in several cancers including melanoma, breast, and lung cancer (163, 164). Recently, KDM5A, and to a lesser extent KDM5B, knockout was found to induce apoptosis in AML (165). In MM, survival analysis in 3 independent patient cohorts of newly diagnosed MM patients identified KDM5B as a bad prognostic factor. Treatment of the MM cell line MM1s with KDOAM-25, a recently developed pan-KDM5 inhibitor, resulted in a G1-phase arrest and a reduction in cell viability. ChIP-sequencing analysis showed that this was accompanied with an increase in H3K4 trimethylation (59). Another promising KDM5 inhibitor, namely compound 33, was recently identified, however this compound was not yet tested in MM (166).

**KDM6A** (also known as UTX) and **KDM6B** (JMJD3) mediate the demethylation of the repressive H3K27me2/3 mark (147). KDM6A/UTX works in concert with other epigenetic modifiers like the HMTs MLL2/3 and HATs P300/CBP, thus mediating transcriptional activation (11). Malignancies can be associated with increases as well as decreases of H3K27 methylation levels and these gains/losses are due to mutations in methyltransferases (like EZH2), demethylases (like UTX), mutations in histone H3 or changes in associated chromatin marks (like MMSET) (103). Loss of UTX, observed in both hematological cancers and solid tumors, is often linked to cell proliferation. In contrast, UTX overexpression in breast cancer has pro-tumoral effects by inducing proliferation and invasion. Therefore, the functional role of UTX seems to be cell context dependent (103). Mutational loss of UTX expression is found in 10% of primary MM samples (12, 167). In HMCL, which are often derived from more aggressive patient cases like extramedullary MM and PCL, the incidence of UTX mutations reaches levels up to 30–40%. To analyze the functional role of UTX in MM, a loss of function study was performed by Ezponda et al. UTX silencing was shown to promote proliferation, clonogenicity, adhesion and tumorigenicity of MM cells. Interestingly, UTX loss was also shown to sensitize MM cells toward EZH2 inhibition. This increased sensitivity was suggested to be linked with reactivation of BCL6 and subsequent repression of IRF4 and c-MYC. Hence, this research suggests a clinical benefit for EZH2i therapy in MM patients harboring an UTX mutation (103). Ohguchi et al. found KDM6B/JMJD3 to be highly expressed in MM and loss of function experiments resulted in the induction of apoptosis (168). In this study, NFkB signaling was suggested to activate KDM6B, which in turn upregulates expression of MAPK-pathway related genes, including ELK and FOS, thus conferring MM survival and growth. Importantly, the KDM6B mediated MAPK pathway activation was found to be demethylase-independent (168). In contrast, KDM6B was recently identified as a tumor suppressor gene that cooperates with TP53 in high-risk patients with a hemozygous deletion of 17p13 (169). Here the tumor suppressive properties of KDM6B were also suggested to be demethylase-independent. Thus, the functional role of KDM6B in MM remains to be further investigated.

## Role of Epigenetic Control in Normal Plasma Cell Differentiation, MM Cell Plasticity and Drug Response

The differentiation of hematopoietic stem cells (HSC) toward antibody producing PCs is also tightly regulated by epigenetic mechanisms and flaws in this epigenetic control can result in various B cell related disorders. In MM, the tumor clone is composed out of different subclones, which differ in maturation stage, clonogenic capacity, and drug sensitivity. Interestingly, a certain plasticity between these subpopulations has been reported and is likely to be controlled by epigenetic mechanisms. Below we will discuss the role of epigenetics in normal PC differentiation, MM cell plasticity and the impact on drug sensitivity.

### Role in Normal Plasma Cell Differentiation

**Figure 2:**
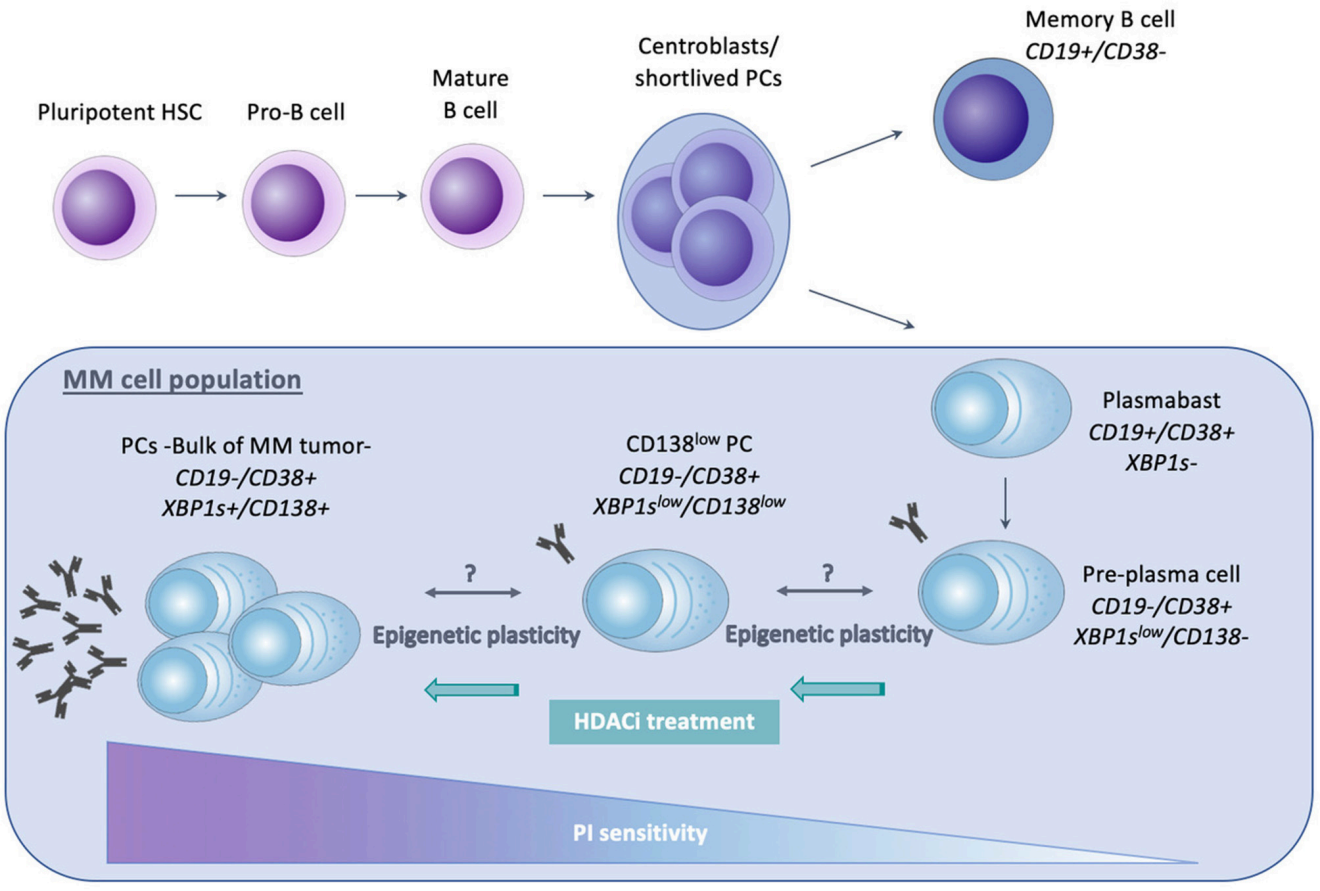
Schematic representation of normal plasma cell differentiation and MM related cellular hierarchy with impact on proteasome inhibitor (PI) sensitivity. The differentiation from pluripotent hematopoietic stem cell (HSC) toward mature, non-dividing and long-living plasma cells (PCs), is shown. The MM clone originates from a post-germinal center long living PC and is believed to be composed of different subpopulations, including CD138^+^/Xbp1s^+^ mature PC (comprising the bulk of the MM clone), CD138^low^/Xbp1s^low^ PC, CD138^−^/Xbp1s^low^ Pre-PC and plasmablasts. These MM cell subpopulations greatly differ in clonogenic capacity, transcriptional profile, and drug sensitivity. The immature, Xbp1s^−/low^ populations lack full secretory status, making them less vulnerable to ER stress and PIs. In MM patients, a bidirectional transition between the CD138^+^ PC and Pre-PC has been proposed and was suggested to be attributed to epigenetic mechanisms, hence referred to as epigenetic plasticity. Epigenetic modulating agents like HDACi have been shown to upregulate Xbp1 and CHOP expression, thus restoring PI sensitivity.

Normal PCs arise from pluripotent HSC, which are present in the adult BM and differentiate into pro-B cells, as illustrated in Figure 2. Maturation of these pro-B cells into naïve B lymphocytes occurs due to heavy and light chain gene rearrangements. Mature B cells from the BM will then migrate toward secondary lymphoid organs, like the lymph nodes and the spleen (170). Upon antigen encounter, these mature B cells will enter the germinal center (GC) and undergo affinity maturation and class switch recombination, forming centroblasts or short-lived PCs. Subsequent maturation and selection cycles in the germinal center give rise to plasmablasts and memory B cells. Upon re-entering the BM, plasmablasts undergo their terminal differentiation toward mature non-dividing and long-living PCs. Their main function is the production and secretion of immunoglobulins (Ig) (171–173). Importantly, B cells with a different maturation status can be distinguished from each other by the presence of specific markers. Both memory B cells as well as plasmablasts express CD19, whereas CD38 is expressed from plasmablast stage onwards. Pre-PCs are characterized by lack of CD19 expression, together with a low expression of CD138 and Xbp1s.

The above described differentiation of mature B cells to PCs is a complex multistep process, involving complex regulatory networks (171). During PC maturation, B cell specific transcription factors important for maintaining a B cell phenotype, like PAX5 and BCL-6, are silenced, whereas PC specific transcription factors like IRF4, Blimp-1 (B-lymphocyte-induced maturation protein, also called PRDM1) and Xbp1 are activated (171, 172). Blimp-1, the so-called master of PC generation, and its transcriptional target Xbp1, play a central role in the development of the unfolded protein response (UPR). Given the highly secretory nature of PCs, these cells are susceptible to endoplasmic reticulum (ER) stress. ER stress, caused by the accumulation of unfolded proteins in the ER, activates the UPR pathway in order to cope with protein accumulation. However, when ER stress exceeds the capacity of the UPR, cells will undergo apoptosis (174). It was shown that ectopic expression of Xbp1 can increase total protein synthesis, the expression of genes involved in secretory pathways and the biogenesis of organelles important in a secretory cell including the ER, mitochondria, and lysosomes. Together, these data clearly demonstrate that Xbp1 expression is important in the differentiation toward a professional secretory cell phenotype (175). Apart from activating Xbp1, Blimp-1 will silence over 250 B cell specific genes, including PAX5 and BCL-6, thus shaping a less proliferative cell population characterized by an increase in Ig synthesis (171, 173).

To silence B cell expression programs, Blimp-1 will interact with various co-repressors of the epigenetic machinery. It was shown that Blimp-1 associates directly with HDACs, recruiting them to the DNA and thereby silencing key B cell specific genes. Blimp-1 and HDAC association was for example shown to decrease histone 3 (H3) acetylation levels associated with the c-MYC promotor, leading to the repression of c-MYC (170, 176). Next to HDACs, Blimp-1 can also regulate gene expression by interacting with HMTs and HDMs, such as G9a and KDM1A/LSD1. Disrupting the interaction between Blimp-1 and LSD1 or silencing of LSD1 reduces the antibody production in secretory cells, thus affecting their functional role (177). Blimp-1 will furthermore recruit G9a to the DNA, leading to an increase in H3K9me3 and repressing several genes, including PAX5 (45, 170, 178, 179). Finally, Blimp-1 was also shown to recruit and bind different complexes like the BAF chromatin remodeling complex, the PRC2 complex, the NuRD complex, the NCoR co-repressor complex and the SIN3 co-repressor complex (180). Together, these data suggest that Blimp-1 acts as a scaffolding protein, thereby recruiting several chromatin and histone modifying components. Recently, Guo et al. showed that EZH2 also plays a role in antibody secreting PCs. EZH2 upregulation was observed in stimulated B cells and especially in PCs, together with increased levels of H3K27me3 in B cell specific promotors. EZH2 deficiency was shown to negatively impact the differentiation into PCs. In addition, these PCs could not repress mature B cell associated transcription programs and produced less antibodies compared to EZH2 expressing counterparts. Furthermore, EZH2 deletions hampered the upregulation of Xbp1 target genes, negatively impacting the expression of the UPR pathway. Together, EZH2 deficiency was found to negatively impact both PC cell number and functionality (86, 170, 181). These observations are in contrast to previous reports stating that EZH2 overexpression is only present in the highly proliferative pre-B and GC B cells (centroblast stage) and suggest an additional functional role for EZH2 in PC function (86, 182). Further supporting the role of epigenetic mechanisms in B cell differentiation, DNA methylation profiling revealed a global shift toward DNA hypomethylation during B cell differentiation, and this occurs mainly at intragenic and intronic regions (183–185). The DNA hypomethylation shift was found to be primarily present in GC B cells, and is suggested to underlie the capacity of these cells to differentiate toward either memory B cells or PCs. Interestingly, the methylation signature of memory B cells and PCs was found to be fairly similar, in contrast to their very different transcriptional profiles (170, 186). This similarity may explain the observation that memory B cells can rapidly differentiate into PCs upon subsequent encounters with the same antigen. Finally, only very recently, RNA sequencing analysis of different stages of PC differentiation, including memory B cells, preplasmablasts, plasmablasts and PCs, identified epigenetic enzymes to be consistently upregulated during PC differentiation, including HMTs (e.g., PRDM1, PRDM15, PRMT7, and SETDB2), the *de novo* DNA methyltransferase DNMT3B, DNA demethylases (e.g., IDH1, IDH2, and TET1) and DNA methylation readers (MBD1 and ZBTB38) amongst others (187).

### Role in Clonal Heterogeneity, Tumor Plasticity and Drug Response in Multiple Myeloma

The MM cell population is thought to originate from a post-germinal center long living PC which retained its capacity to proliferate (10). The bulk of the MM cells consist of mature CD19-, CD138+, and Xbp1s expressing cells (172). However, emerging evidence has shown that within the MM population, different subpopulations exist which differ in propagating (clonogenic) potential, maturation stage, transcriptional profile, and drug sensitivity (10, 172, 188–190). As shown in Figure 2, these MM populations include both cells with a B cell and PC phenotype. However, only the latter seem to have myeloma-propagating properties. Clonogenic CD19+ B cells for example are unable to propagate MM after implantation *in vivo*, in contrast to the pre-PC (CD19-CD138-), CD138low and CD138+ PCs (172, 191). Interestingly, it was suggested that within the clonotypic PC fraction differentiation programs might be reversed: in xenograft models where CD138+ PCs were engrafted, pre-PCs could be isolated and vice versa. This observed bidirectional transition was suggested to be most likely regulated through epigenetic mechanisms and was referred to as epigenetic plasticity. In support of this, pre-PCs were shown to be enriched in epigenetic regulators compared to PCs. These regulators include HMTs belonging to the PRC2, components of the MLL transcriptional activating complex, demethylases such as KDM5C/D, HATs, and HDACs (188). Moreover, as previously mentioned, Aggire et al. recently reported hypermethylation of enhancer regions of B cell specific genes and transcription factors such as PAX5, BATF, and STAT5, leading to their downregulation. These enhancers are highly methylated in stem cells, and demethylation occurs during normal B cell differentiation into PCs (33). This suggests that MM cells either regain stem cell like epigenetic features or that they are able to retain the features of a MM stem cell progenitor.

**Figure 3:**
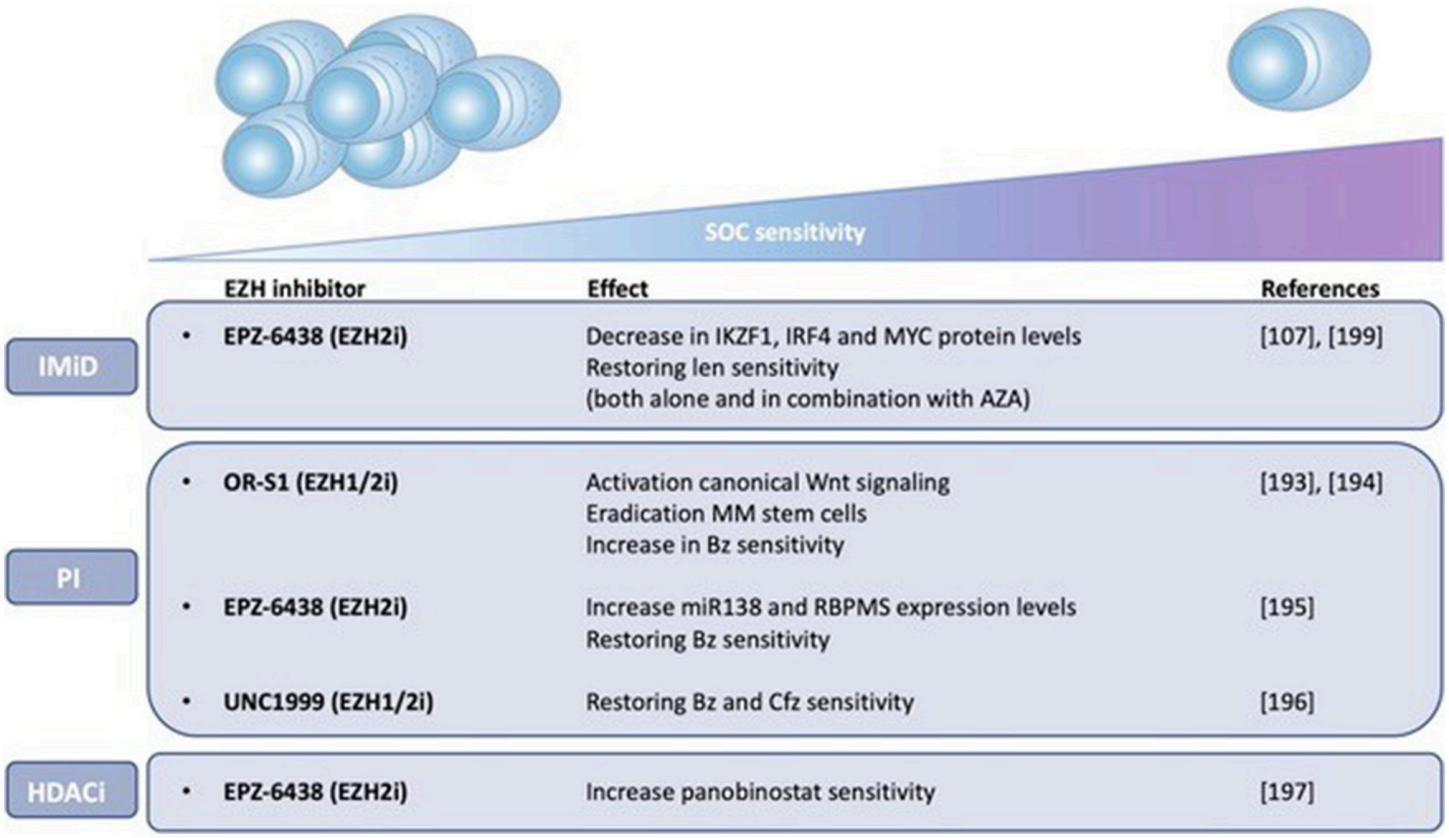
EZH2 mediates drug responses in MM. The impact of EZH1/2 inhibition on MM sensitivity toward standard-of-care (SOC) agents, including immunomodulatory drugs (IMiDs) and proteasome inhibitors (PIs), and histone deacetylase inhibitors (HDACi) is shown. Possible underlying mechanisms of action are described if known. Len (lenalidomide), Bz (bortezomib), Cfz (carfilzomib), AZA (5-azacytidine).

Importantly, the pre-PCs and clonogenic B cell populations are more quiescent and drug resistant than PCs and are thus believed to play a role in clinical drug resistance and relapse (172, 188). Leung-Hagesteijn et al. found that the immature subpopulations are intrinsically PI resistant and persist in bortezomib treated patients. As a possible mechanism for the PI resistance, the authors proposed a lower activity of the IRE-XBP1s axis in the plasmablasts, pre-PCs (CD19-CD138-) and CD138low PCs. PIs create an accumulation of misfolded proteins in the aggresome, causing lethal ER stress. This explains the vulnerability of secretory cells toward these agents. However, the immature Xbp1-/low cell populations are less proliferative and lack full secretory status, making them less vulnerable to lethal ER stress (172, 189). Together, these data give a possible explanation for the failure of PI based therapy, both bortezomib and carfilzomib, in curing MM (172, 189). Given the presumed role of epigenetic mechanisms in the proposed bidirectional pre-PC/PC transition, it might be plausible to assume that epigenetic therapies might help overcome PI resistance in MM by inducing epigenetic reprogramming of the CD138-/CD138low subpopulations toward an Xbp1 positive state (172). In support of this, HDAC1 was found to be highly expressed in CD138- propagating cells causing Xbp1 and CHOP repression, thus reducing sensitivity toward PIs (188, 189). Treatment of these cells with HDAC inhibitors was shown to upregulate Xbp1 and CHOP expression and therefore restore PI sensitivity (58, 192). These data offer a possible explanation (apart from the effect on HDAC6) for the favorable results of the aforementioned PANORAMA (PANobinostat ORAl in Multiple myelomA) trials. As shown in Figure 3, several studies have also implicated EZH2 in mediating drug responses in MM. Nakagawa et al. for example found EZH1/2 expression levels to be higher in a MM side population thought to comprise MM stem cells, suggesting that EZH1/2 expression plays a role in maintaining MM stemness. The dual EZH1/2 inhibitor OR-S1 eradicated these MM stem cells and activated canonical Wnt signaling, thus inhibiting self-renewal and differentiation of HSC (193). The elimination of stem cell-like MM cells upon EZH2 targeting (both alone and in combination with bortezomib), was also confirmed in a second study (194). Recently, Rastgoo et al. reported the importance of a EZH2/miR-138 axis in MM drug resistance. EZH2 overexpression was shown to confer drug resistance toward anti-MM agents (including bortezomib) and associate with a bad prognosis. Mechanistically, EZH2 overexpression was found to silence miR-138 and RBPMS (RNA-binding protein with multiple splicing) in drug resistant cells and increasing RBPMS levels by using EZH2i or miR-138 mimics restored bortezomib sensitivity. Moreover, combination therapy with bortezomib and the EZH2i EPZ-6438 significantly delayed tumor growth in a xenograft model compared to single agent therapy (195). Rizq et al. also found MM patients with high EZH2 expression levels to be more resistant to bortezomib treatment. In concordance, combining the dual EZH1/2 inhibitor UNC1999 with bortezomib or carfilzomib significantly enhanced the anti-MM effects of these PIs, both *in vitro* and *in vivo*. Of note, these effects were superior to the results observed when combining bortezomib with GSK126, a selective EZH2 inhibitor, indicating that targeting EZH1 and EZH2 simultaneously is more effective (196). Together, the studies described above highlight the potential of EZHi in eradicating MM stem cells and overcoming PI resistance. Next to bortezomib, a recent study found that pretreatment with EZH2i also sensitizes MM cells to the HDACi panobinostat *in vitro* (197). In addition, pretreatment of MM cells with the EZH2i EPZ-6438 restored sensitivity toward the IMiD lenalidomide. As a possible underlying mechanism of action, the significant decrease in IKZF1, IRF4, and MYC protein levels upon combination treatment was suggested (107). In support of this, we showed only very recently that HDACi and DNMTi combination treatment decreases IRF4 and MYC levels and induces a more mature BMPC gene expression profile in myeloma cell lines. Moreover, we constructed a gene-expression based score to predict patient outcome and MM sensitivity toward HDACi/DNMTi combination treatment. Patients with al low combo score were characterized by a mature BMPC gene signature, whereas patients with a high combo score were characterized by a proliferating and MYC-associated gene signatures and worse overall survival. Nevertheless, these high-risk patients were found to display a higher sensitivity of their MM cells to HDACi/DNMTi combination treatment. Thus, our data suggest a therapeutic benefit for combining IMiD therapy with DNMTi/HDACi in high-risk MM patients with a high combo score (198). Dimopoulos et al. also found that simultaneous targeting of DNMTs and EZH2 overcomes IMiD resistance in MM. IMiD resistant cells were characterized by an increase in genome-wide DNA methylation levels and a reduction in chromatin accessibility and gene expression levels. Treatment with AZA and EPZ-6438 reversed these observed changes in chromatin structure and resensitized the cells to IMiDs independently of cereblon. Together, this study suggests that IMiD-acquired resistance in MM is mainly epigenetically mediated and that combination with specific epidrugs could restore IMiD sensitivity (199). Finally, next to the above described role of EZH2 in IMiD and PI resistance, it should be mentioned that Kikuchi et al. also described a correlation between phosphorylation-mediated inactivation of EZH2 and cell adhesion-mediated drug resistance (CAM-DR) against doxorubicin and the alkylating agent 4-OHCY in MM. IGF-1R and PI3K/Akt inhibitors reversed this CAM-DR by blocking the IGF-1 mediated EZH2 phosphorylation (200). However, in this study only *in vitro* data was included, performed on merely 2 HMCL.

## Conclusion and Future Perspectives

Although cancer is typically considered a genetic disease, multiple lines of evidence have shown that defects in the epigenetic machinery are equally as important in cancer onset and progression. In fact, epigenetic lesions have been proposed to contribute to many classical hallmarks of cancer, including but not limited to genomic instability, sustained proliferation, invasion and metastasis, evading the immune system and metabolic dysregulation, and might even be considered as a new, additional cancer hallmark. In MM, mutations in DNA methylation and histone acetylation and methylation modifiers and the associated alterations in chromatin states have also repeatedly been shown to play prominent roles in genomic instability, sustained proliferation and drug resistance. Consequently, targeting these epigenetic regulators using e.g., pan-HDACi and EZH2i induce potent anti-MM effects both in preclinical studies and clinical trials, especially in combination with PIs and IMiDs. Recent evidence is also suggesting that apart from inducing direct anti-tumor effects, these epigenetic modulating agents might also reprogram the immature (Xbp1s-/low) MM subpopulations toward a bulk of mature Xbp1s^+^ PCs with a higher drug sensitivity, thus limiting the survival of drug resistant clones and chance to relapse. However, although targeting epigenetic modifiers in MM therapy looks promising, additional (pre)clinical studies are still mandatory before these agents can be fully implemented into daily clinical practice. Firstly, based on the findings that epidrugs might induce maturation of the Xbp1s-/low populations, the efficacy of combination therapies should also be evaluated in newly diagnosed MM patients instead of relapsed/refectory MM patients. Secondly, as the pan-HDACi and -DNMTi currently used in clinic are often associated with high toxicity profiles, new regimens combining more selective epigenetic modifying agents, such as EZH2i and other specific HMTi, with standard of care agents should be explored to improve tolerability, while maintaining efficacy. Finally, given the high epigenetic heterogeneity, MM patients could greatly benefit from combined genetic and epigenetic profiling. Sequencing of patient samples during disease progression (that is at the moment of diagnosis, during therapy and upon relapse) could lead to the identification of novel epigenetic targets and biomarkers and pave the way for personalized treatment strategies.

## Author Contributions

EDS, HL, and EDB developed the design and arguments for the paper and drafted the manuscript. KM, EM, KD, and KV revised the manuscript.

### Conflict of Interest Statement

The authors declare that the research was conducted in the absence of any commercial or financial relationships that could be construed as a potential conflict of interest.
